# Antisense oligonucleotides and all-trans retinoic acid have a synergistic anti-tumor effect on oral squamous cell carcinoma

**DOI:** 10.1186/1471-2407-8-159

**Published:** 2008-06-03

**Authors:** Qin Xu, Zhiyuan Zhang, Ping Zhang, Wantao Chen

**Affiliations:** 1 Department of Oral and Maxillofacial Surgery, Ninth People's Hospital, Shanghai Jiao Tong University School of Medicine, Shanghai Key Laboratory of Stomatology, 639, Zhizaoju Road, Shanghai 200011, PR China; 2 Department of Thoracic/Head and Neck Medical Oncology, M.D. Anderson Cancer Center, The University of Texas,1515 Holcombe Boulevard, Box 432, Houston, Texas, 77030, USA

## Abstract

**Background:**

Antisense oligonucleotides against hTR (As-ODN-hTR) have shown promising results as treatment strategies for various human malignancies. All-trans retinoic acid (ATRA) is a signalling molecule with important roles in differentiation and apoptosis. Biological responses to ATRA are currently used therapeutically in various human cancers. The aim of this study was to evaluate the anti-tumor effects of As-ODN-hTR combined with ATRA in vivo.

**Methods:**

In situ human oral squamous cell carcinoma (OSCC) models were established by subcutaneous injection of Tca8113 cells. Mice were treated with sense oligonucleotides against hTR(S-ODN-hTR) alone, As-ODN-hTR alone, ATRA alone, As-ODN-hTR plus ATRA, or S-ODN-hTR plus ATRA. Tumor size and weight were assessed in the mice. Telomerase activity was detected by a TRAP assay, apoptotic cells were evaluated with a Tunel assay, the expression of apoptosis-related proteins (Bcl-2 and Bax) was evaluated by immunohistochemistry and ultrastructural morphological changes in the tumor specimen were examined.

**Results:**

Both As-ODN-hTR and ATRA can significantly inhibit tumor growth in this OSCC xenograft solid-tumor model, and the combination of the two agents had a synergistic anti-tumorogenic effect. We also demonstrated that this anti-tumor effect correlated with inhibition of telomerase activity. Furthermore, significant increases in the number of apoptotic cells, typical apoptotic morphology and a downregulation of the anti-apoptotic protein, bcl-2 were observed in the treated tissues.

**Conclusion:**

The combination of As-ODN-hTR and ATRA has a synergistic anti-tumor effect. This anti-tumor effect can be mainly attributed to apoptosis induced by a decrease in telomerase activity. Bcl-2 plays an important role in this process. Therefore, combining As-ODN-hTR and ATRA may be an approach for the treatment of human oral squamous cell carcinoma.

## Background

Head and neck cancer affects more than half a million patients worldwide each year. Despite improvements in surgery, chemotherapy, and radiation therapy, overall survival has not improved over the past 30 years. Therefore, the development of more effective therapeutic tools and strategies is urgently required.

Telomerase is a ribonucleoprotein that adds telomeric repeats (TTAGGG)n to the ends of chromosomes. Telomerase expression is now believed to be an important step in the development of cellular immortality and oncogenesis. Recently, it has been shown that approximately 90% of malignant tumors of various origins express telomerase, whereas most normal tissues do not [1]. The human telomerase holoenzyme consists of the human telomerase RNA template subunit (hTR), the human telomerase reverse transcriptase (hTERT) and a number of associated proteins. Both the telomerase RNA component hTR and hTERT are essential for telomerase function, therefore these components may be good candidates for targeted therapy of malignant tumors [2]. Strategies using antisense oligonucleotides (As-ODN) against hTR (As-ODN-hTR) have shown promising results as treatment strategies for various human malignancies [3,4]. However, since antisense therapy was unable to completely inhibit telomerase activity and directly kill tumor cells *in vivo*, it was suggested that this strategy would have to be combined with another approach in order to provide an adequate level of therapy.

All-trans retinoic acid (ATRA), a vitamin A derivative, is a signalling molecule with important roles in growth, differentiation, and apoptosis in a variety of normal adult and embryonic tissues. These functions of ATRA are partly mediated by mechanisms that inhibit telomerase activity [5]. Biological responses to ATRA are currently used therapeutically in various human cancers including leukemia, skin cancer, cervical cancer and neuroblastoma [6-8]. The ability of ATRA to modulate differentiation, apoptosis, and proliferation of tumor cells through inhibition of telomerase has been suggested to explain its efficacy against tumors [9,10]. Based on this hypothesis, we postulated that the combination of As-ODN-hTR and ATRA therapy might have synergistic anti-tumor effects.

We have previously reported that the combination of oligonucleotide (a synthesized 19-mer) As-ODN-hTR treatment with ATRA had a cytotoxic effect on oral squamous cell carcinoma (Tca8113) *in vitro*, in a synergistic manner [11]. The aim of this study was to evaluate the *in vivo *anti-tumor effects of As-ODN-hTR combined with ATRA in an *in situ *human oral squamous cell carcinoma (OSCC) model in mice.

## Methods

### Antisense oligodeoxynucleotides and drugs

The antisense phosphorothioate oligonucleotide (As-ODN-hTR) targeting bases 65 to 46 of hTR mRNA (5'-cccttctcagttagggttag-3') and the hTR-Sense phosphorothioate oligonucleotide (S-ODN-hTR) (5'-ctaaccctaactgagaaggg-3') were synthesized with partial thio-modifications by Shanghai ShenBioColor Company. The sense sequence was used as a control. ATRA (Sigma, U.S.A.) was dissolved in seed oil at concentration of 3 mg/ml and shielded from the light with aluminium foil until used.

### Cell culture and experimental animals

Tca8113, a cell line derived from a human primary oral squamous cell carcinoma, was maintained in RPMI-1640 with 10% fetal bovine serum (FBS) at 37°C in an atmosphere with 5% CO_2_.

The virgin female nu/nu BALB/c mice (4 weeks old; 19 ± 2 g) used in these experiments were obtained from the Shanghai Laboratory Animal Center of China Academy of Sciences. All animal experiments were carried out according to the standards of animal care as outlined in the Guide for the Care and Use of Experimental Animals of Medical College of Shanghai Jiaotong University. The human OSCC tumor model has been previously described [12]. Briefly, the Tca8113 cells were harvested by trypsinization and resuspended at a concentration of 1 × 10^7 ^cells/ml. The mice were then subcutaneously implanted with 1 × 10^6 ^cells into both the right and left hind limbs using a 27 gauge needle/0.5 ml tuberculin syringe. Seven days later, when tumor nodules were palpable, mice were randomly assigned to one of six groups: S-ODN-hTR alone (S group); As-ODN-hTR alone (As group); ATRA alone (ATRA group); S-ODN-hTR plus ATRA (S/ATRA group); As-ODN-hTR plus ATRA (As/ATRA group) and no treatment (control group). There were five mice in each group.

### Treatment in Nude Mice

Fourteen days after tumor inoculation, when the subcutaneous tumor had grown to a visible size, the different treatments were administered to the established tumor. The As group received an intra-tumoral injection of As-ODN-hTR DNA (1 mg/kg in a volume of 50 μl) three times a week for four weeks. The S group received an intra-tumoral injection of S-ODN-hTR DNA (1 mg/kg in a volume of 50 μl) three times a week for four weeks. Mice of ATRA group were gavaged ATRA daily at a dose of 15 mg/kg/d for four weeks. The As/ATRA group received both an intra-tumoral injection of As-ODN-hTR DNA and ATRA administration. The S/ATRA group received both intra-tumoral injection of S-ODN-hTR DNA and ATRA administration. The control group received intra-tumoral injection of the same volume of phosphate buffered saline. At the study endpoint, mice were sacrificed by cervical dislocation and the tumors were removed and weighed. Tumor sizes were monitored with calipers twice a week, and the tumor volume (V, mm^3^) was calculated as (L × W^2^)/2, where L = length (mm) and W = width (mm). The percentage of tumor growth inhibition was calculated as: Inhibitory rate (%) = (Weight_control_-Weight_treat_)/Weight_control _× 100.

### Tissue specimens

A total of 60 tissue specimens were obtained after sacrificing the animals. The samples were immediately cut into three parts. One part was frozen in liquid nitrogen, and stored at -80°C until examined. The second part was fixed in 10% buffered formalin, and fragments were embedded in paraffin before sections (4 μm) were cut. The third part was fixed with 4% glutaraldehyde in phosphate buffer for further transmission electronic microscopy analysis. Care was taken to use separate instruments to harvest each specimen in order to avoid cross contamination.

### Terminal deoxynucleotidyl transferase (TDT) dUTP nick end labelling (TUNEL)

Evaluation of apoptotic cells was performed using a Tunel assay, using an *in situ *Cell Death Detection kit (Oncor, U.S.A.). Briefly, the paraffin sections were treated with 20 μg/ml proteinase K for 15 min and blocked with 3% hydrogen peroxide in methyl alcohol for 10 min. After washing in PBS, sections were incubated with equilibration buffer, followed by TDT enzyme in a humidified chamber at 37°C for 1 hr, and then a stop/wash buffer was applied for 30 min at 37°C. Finally, the sections were incubated with peroxidase labelled anti-digoxigenin antibody for 30 min at room temperature. Positive cells were visualized using a diaminobenzidine substrate and counterstained with hematoxylin. TUNEL positive cells showed brown staining of the nucleus suggestive of internucleosomal DNA cleavage.

### Immunohistochemistry (IHC)

IHC for Bcl-2 and Bax was performed using standard methods [13]. Endogenous peroxidase activity was blocked by treatment with 3% hydrogen peroxide in PBS for 30 min. The specimens were rinsed in PBS. The tissue sections were stained with a mouse monoclonal anti-Bcl-2 antibody (DAKO, Denmark) and a rabbit polyclonal anti-Bax antibody (Santa Cruz, U.S.A.). The sections were incubated overnight at 4°C (dilution of primary antibodies 1:50). The bound antibody was detected with a secondary biotinylated antibody for 30 min at room temperature (1:200 dilution) and visualized using diaminobenzidine as a chromogenic substrate. The sections were then counterstained with hematoxylin.

### Transmission electron microscopy (TEM)

For ultrastructural examination, ultra-thin sections were cut on a Reichert Ultracut ultramicrotome (Mager Scientific Inc., U.S.A.), mounted on 150-mesh copper grids, and post-stained in uranyl acetate and Reynold's lead citrate. Sections were photographed using a transmission electronic microscope (PHILIP CM-120).

### Evaluation of TUNEL and IHC Staining results

The levels of TUNEL and IHC staining were quantified using a semi-automated computerized image analysis system. The image analysis software program used was Image Pro Plus 6.0, which has previously been successfully applied to analyze histological sections [14,15]. The Integrated Optical Density (IOD, equal to Area × Average Optical Density) of positive staining was calculated for each tissue section. The average IOD scores were calculated from triplicate values from each section. The image analysis was performed by three pathologists blinded to the treatment group.

### Measurement of telomerase activity by TRAP assay

Batches of frozen biopsies were thawed on ice and homogenized in telomerase assay buffer. The protein concentration of the supernatant was determined using a Protein Assay Kit (Pierce Biotechnology, U.S.A.). 1 μg of protein was then analyzed using a TRAP (Telomeric Repeat Amplification Protocol) assay (Roche, Germany) to quantify telomerase activity. Briefly, this protocol consisted of incubating the protein lysate with an artificial telomerase template, allowing the telomerase to extend the template. The elongated products were then amplified by PCR using biotin-labelled telomere-specific primers. Aliquots of the PCR products were denatured, hybridized to DIG-labelled sequence-specific probes and bound to streptavidin-coated microtiter plates. The products were detected with peroxidase conjugated anti-DIG antibodies and TMB as a substrate. The optical density (OD) was read at 450 nm against a blank (reference wavelength 620 nm). Each sample was measured twice.

### Statistical analysis

Results were expressed as mean ± standard error (SE). Statistical differences between groups were evaluated by one-way analysis of variance (ANOVA), and two-way ANOVA was used to determine interactions between As-ODN-hTR and ATRA. All data were analyzed with SPSS 10.0 software. *P *< 0.05 was considered statistically significant. The combined anti-tumor effect of As-ODN-hTR and ATRA was evaluated using the fractional product method. Fractional tumor volume (FTV) relative to controls was calculated as the ratio between the mean tumor volume of the experimental groups and the mean tumor volume of the control group. The expected FTV of the combination is the product of the mean FTV of the two treatments. The ratio was obtained by dividing the expected FTV by the observed FTV of the combination. An FTV ratio of >1 indicates a synergistic effect, and a ratio of <1 indicates a less than additive effect.

## Results

### Effects of different antisense ODNs on tumor growth

**Figure 1:**
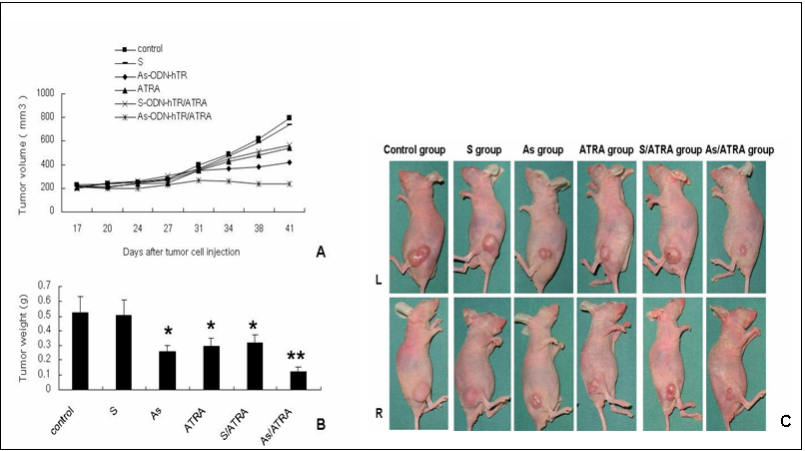
**The effect of the treatment with antisense oligonucleotide against hTR(As-ODN-hTR), all-trans retinoic acid (ATRA) on the growth of OSCC tumor xenografts in nude mice**. A, tumor growth curve is showed. B, tumor weight is given as the mean ± SE (bars) (n = 10). C, representative mice and tumors in each experimental group is shown at the end of treatment (L = left side, R = right side). Both As-ODN and ATRA treatment resulted in an inhibition of tumor volume and tumor weight as compared to control group. * *P *< 0.01, one-way ANOVA. In addition, highly significant interactions exist between hTR As-ODN and ATRA. * * F = 10.743, *P *= 0.002, two-way ANOVA. The combination of As-ODN and ATRA resulted in a significant enhancement of the reduction in tumor growth when compared with monotherapy of As-ODN or ATRA alone (*P *< 0.01).

In the current study, we tested the influence of S-ODN, As-ODN, ATRA, As-ODN plus ATRA, or S-ODN plus ATRA on the growth rate of Tca8113 tumors *in vivo*. As shown in Figure 1, at the end of treatment the mean tumor size and tumor weight were 740.8 ± 147.4 mm^3 ^and 0.504 ± 0.101 g in the S group, 422.7 ± 77.8 mm^3 ^and 0.26 ± 0.043 g in the As group, 544.7 ± 93.3 mm^3 ^and 0.294 ± 0.054 g in the ATRA group, 564.9 ± 107.8 mm^3 ^and 0.319 ± 0.054 g in the S/ATRA group and 240.4 ± 40.9 mm^3 ^and 0.123 ± 0.031 g in the As/ATRA group, respectively. In contrast, tumor volume and tumor weight in the control group were 796.2 ± 133.7 mm^3 ^and 0.522 ± 0.109 g, respectively. Both As-ODN and ATRA treatment resulted in a reduction in tumor volume and tumor weight when compared to the control group (*P *< 0.01, one-way ANOVA). However, no tumor inhibition was observed in the S-ODN treated group.

Treatment of mice with a combination of As-ODN and ATRA resulted in a significant enhancement of the reduction in tumor growth when compared with therapy with either As-ODN (*P *< 0.01) or ATRA alone (*P *< 0.01). Statistical analysis indicated that a highly significant interaction exists between As-ODN-hTR and ATRA (F = 10.743, *P*= 0.002, two-way ANOVA). The anti-tumor efficacy of As-ODN in combination with ATRA was assessed by a fractional product method. From day 10 to day 28 after treatment, the ratio between the expected FTV and the observed FTV of the combination ranged from 1.05 to 1.27 (Table 1), clearly indicating synergy between As-ODN-hTR and ATRA treatment.

When compared with the control group, the inhibition on the rate of tumor growth was 50.2%, 43.7%, 38.9%, and 76.4% in the As group, ATRA group, S/ATRA group, and As/ATRA group, respectively. The greatest growth inhibition occurred in the combination treated group.

**Table 1:** Effect of combined treatment of As-ODN-hTR and ATRA on the growth of tumors.

FTV relative to untreated controls^a^					
			Combination treatment		
				
Treated Day^b^	As-ODN-hTR	ATRA	Expected^c^	Observed	Ratio of expected FTV/observed FTV^d^

3	0.972	0.894	0.869	0.945	0.92
7	0.848	0.883	0.749	0.841	0.891
10	0.957	0.922	0.882	0.794	1.111
14	0.99	0.9	0.89	0.844	1.055
17	0.892	0.877	0.782	0.681	1.149
21	0.753	0.879	0.662	0.531	1.246
24	0.618	0.783	0.484	0.379	1.277
28	0.53	0.684	0.363	0.302	1.202

^a ^FTV, fractional tumor volume, calculated as mean tumor volume experimental/mean tumor volume control.

^b^Day after tumors treated with agents.

^c^Mean FTV of As-ODN-hTR X mean FTV of ATRA.

^d^Obtained by dividing the expected FTV by the observed FTV. A ratio of >1 indicates a synergistic effect, and a ratio of <1 indicates a less than additive effect.

### Apoptosis detection

**Figure 2:**
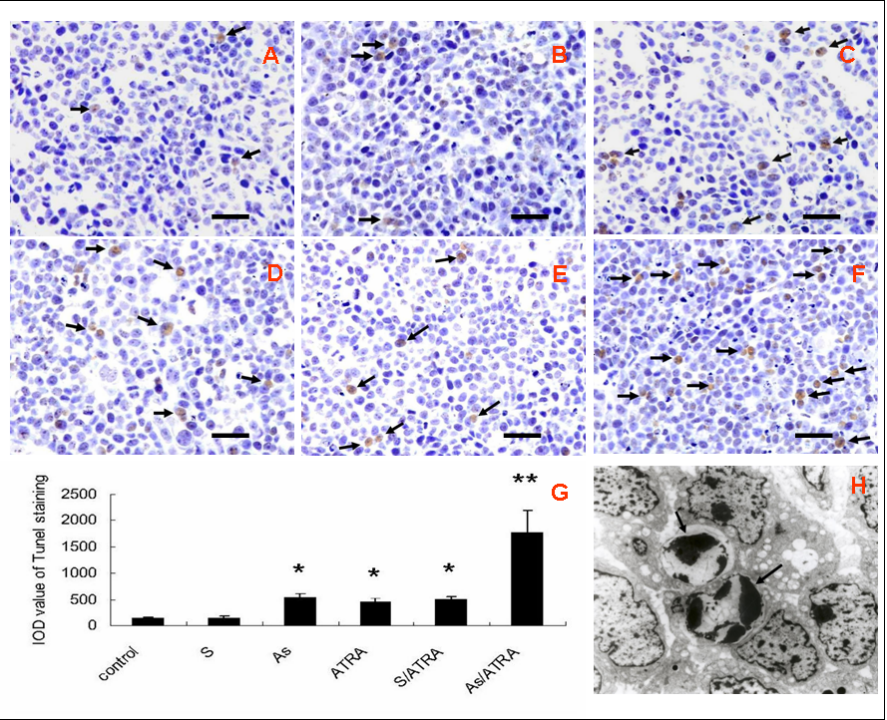
**Cell apoptosis in treatment groups and control group**. (A-F) Apoptotic cells were revealed by TUNEL-histochemistry assay. Representative microphotographs of Tunel staining of (A) control group, (B) S group, (C) As group, (D) ATRA group, (E)S/ATRA group and (F) As/ATRA group. Arrows indicate positive cells. Scale bar = 50 μm. The average Integrated Optical Density (IOD) of each group was performed by a computer-assisted quantitation. (G) IOD data is shown as the mean ± SE (n = 10). The average IODs in As-ODN and ATRA treated groups were significantly increased as compared to S-ODN group and control group. * *P *< 0.01, one-way ANOVA. Significant interaction was observed between As-ODN and ATRA treatment. * * F = 45.918, *P *< 0.01, two-way ANOVA. (H) Electron microscopy showed typical feature of apoptotic cell in As-ODN and ATRA treatment groups. Whereas, no ultrastructural changes were observed in S-ODN group and control group (data not shown).

Cell apoptosis was detected by TUNEL staining and ultrastructural morphological changes were examined by transmission electron microscopy (TEM). Assessment of cell death by TUNEL histochemistry revealed a dramatic increase in TUNEL-positive cells in the As-ODN and ATRA treated groups, and this increase was extremely high in the As/ATRA combination group compared to the S and control groups. (Fig. 2A–F). The average Integrated Optical Density (IOD) of each group in the TUNEL test was obtained by a computer-assisted quantification method. The average IODs in the As-ODN and ATRA treated groups were significantly increased compared to the S group and control group (*P *< 0.01, one-way ANOVA). A significant interaction was observed between the As-ODN and ATRA treatments (F = 45.918, *P *< 0.01, two-way ANOVA) (Fig. 2G). From the results of transmission electron micrography, features associated with the typical morphology of apoptotic squamous carcinoma cells, such as nuclear condensation and fragmentation were observed in cells from the As-ODN and ATRA treated groups (Fig. 2H).

### Semi-quantification of Telomerase Activity

**Figure 3:**
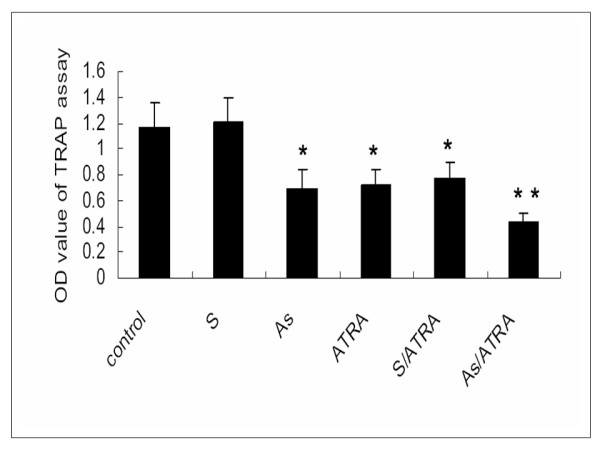
**Telomerase activity of each group was detected by TRAP assay, a PCR-based semiquantitative method**. The average OD values of TRAP assay were shown as the mean ± SE (n = 10). Telomerase activity was significantly higher in S-ODN group and control group compared with other treatment groups. * *P *< 0.01, one-way ANOVA. A significant interaction was observed between As-ODN-hTR and ATRA treatment. * * F = 4.507, *P*= 0.041, two-way ANOVA.

Using a TRAP assay, we found that telomerase activity was significantly higher in the S group and control group compared to the other treatment groups (*P *< 0.01, one-way ANOVA). A significant interaction was observed between As-ODN-hTR and ATRA treatment (F = 4.507, *P *= 0.041, two-way ANOVA). (Fig. 3).

### Expression of Bcl-2 and Bax

**Figure 4:**
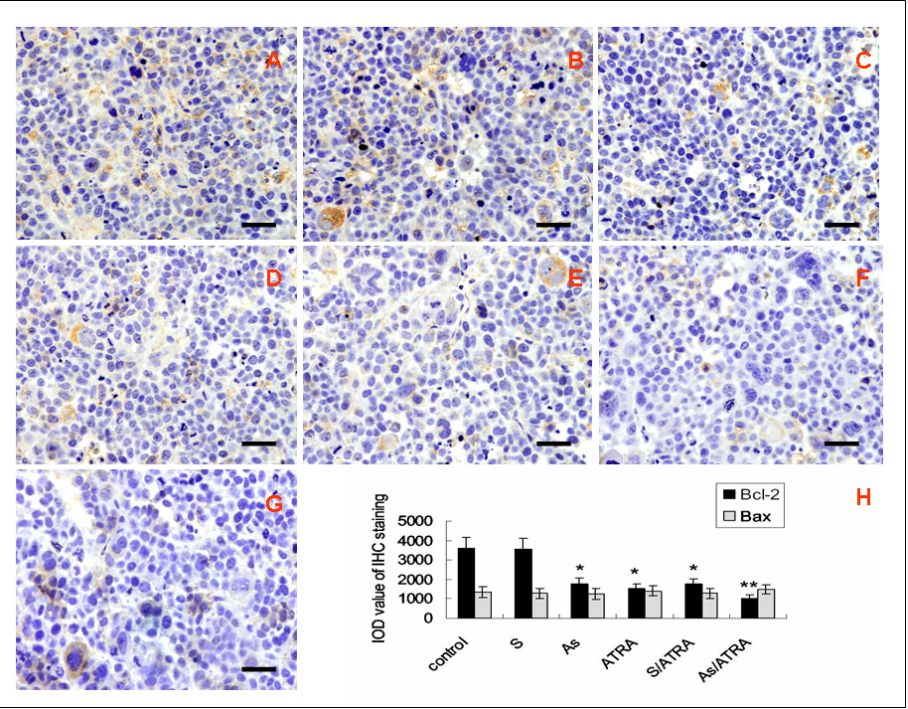
**Immunohistochemical staining for Bcl-2 (A-F) and Bax (G) expression in treatment groups and control group**. Sections showed strong Bcl-2 cytoplasmic staining of tumor cells in (A) control group and (B) S-ODN group, weak Bcl-2 cytoplasmic staining in (C) As group, (D) ATRA group and (E) S/ATRA group, extremely weak Bcl-2 staining in (F) As/ATRA treated group. (G) Similar moderate Bax cytoplasmic staining of tumor cells in all treatment groups. Representative section of As/ATRA group is shown. Scale bar = 50 μm. Computer-assisted quantitation of immunohistochemical staining was performed. (H) The average IODs of Bcl-2 and Bax were shown as the mean ± SE (n = 10). The average IODs in As-ODN and ATRA treated groups were significantly decreased as compared to S-ODN group and control group. * *P *< 0.01, one-way ANOVA. Significant interaction was observed between As-ODN-hTR and ATRA treatment. * * F = 35.836, *P *< 0.01, two-way ANOVA. There was no significant difference of Bax expression in all treatment groups.

Both Bcl-2 and Bax specific immunostaining were restricted to the cytoplasm. Bcl-2 immunostaining was predominantly found in the S group and the control group (Fig. 4A–B). However, the expression of Bcl-2 in the As-ODN and ATRA treated groups was very low, and was extremely low in the As/ATRA combination group (Fig. 4C–F), The average IODs in the As-ODN and ATRA treated groups were significantly decreased compared to the S group and control group (*P *< 0.01, one-way ANOVA).(Fig. 4H). A significant interaction was observed between As-ODN-hTR and ATRA treatment (F = 35. 836, *P *< 0.01, two-way ANOVA). However, the expression of Bax was similar in all experimental groups (Fig. 4G).

## Discussion

It has been hypothesized that in the combination of As-ODN-hTR and ATRA may have an additive anti-tumor effect. However, previous reports that may relate to this hypothesis are very limited. In our previous study, we reported that the combination of As-ODN-hTR and ATRA could synergistically inhibit telomerase activity in OSCC (Tca8113) and induce cell growth arrest [11]. The purpose of this study was to examine the combined anti-tumor effects of As-ODN-hTR and ATRA treatment *in vivo*.

In this study, the effects of As-ODN-hTR and ATRA on the growth of human OSCC xenografts were evaluated. It was demonstrated that both As-ODN-hTR and ATRA have anti-carcinogenic effects in our tumor model. However, controlled S-ODN-hTR treatment had no effect on tumor growth, indicating that the observed inhibitory effect was sequence specific. Moreover, a critical observation was that there was a clear synergistic anti-tumor efficacy of combination treatment with As-ODN-hTR and ATRA, compared with monotherapy. This marked interaction effect resulted in the highest rate of inhibition of tumor growth in all experimental groups, strongly supporting our hypothesis that these two agents have a synergistic anti-tumor effect, which is consistent with our previous findings *in vitro *[11].

In an effort to determine the mechanism behind this synergistic anti-tumor efficacy, the telomerase activity and levels of cellular apoptosis in each group was investigated.

Telomerase activity was investigated using a TRAP assay. The telomerase activity was decreased in the As-ODN-hTR and ATRA treatment groups compared to the S and control groups. Although a potential limitation of oligonucleotide monotherapy is bioavailability to tumor tissues [16], our results demonstrated that intra-tumoral injection of As-ODN-hTR thrice a week, combined with daily ATRA treatment could decrease telomerase levels by up to 76.4% compared with control tumors. The combination of As-ODN-hTR and ATRA had a greater effect on telomerase activity inhibition than either of the agents used alone. This result is consistent with the observations of tumor growth inhibition. The correlation between telomerase activity and tumor inhibition suggests that the ability of As-ODN-hTR and ATRA to inhibit tumor growth is due to their suppression of telomerase activity. Many studies have suggested that the *hTERT *gene may be the primary target of ATRA regulation of cellular differentiation [9,10]. The As-ODN-hTR used in the present study was designed against the hTR component of telomerase. Therefore, the synergistic telomerase suppression effect observed after combination treatment may be due to the fact that the two agents simultaneously act against these two major telomerase components. Further studies are required to determine the mechanism by which As-ODN-hTR and ATRA operate synergistically. Moreover, telomerase inhibitors dependent on mechanisms of telomere attrition would need to be continuously administered on a long-term basis for weeks to months in order to produce anti-tumor effects [3]. This may be the reason that the observed synergistic anti-tumor effect occurred after 10 days administration of the combination treatment.

In addition, apoptotic cells, expression of proteins related to apoptosis (Bcl-2/Bax) and ultrastructural morphological changes were also observed in the present study. Our data showed that As-ODN-hTR and ATRA treatment increased apoptotic cell death, and the most significant increase in apoptosis was noted in the combination treated group, as seen by TUNEL staining. Transmission electron micrography revealed cells with morphologies typical of apoptotic squamous carcinoma cells in all treatment groups, whereas no ultrastructural changes were observed in the control group. In the combination of As-ODN-hTR and ATRA, treatment appears to effectively trigger apoptosis *in vivo*. Therefore, the possible mechanism underlying inhibition of tumor cell growth in our study may occur via apoptosis.

Bcl-2 family proteins are key regulators of mitochondrial integrity and comprise both pro- and anti-apoptotic proteins [17]. Bcl-2 is considered to be an anti-apoptotic protein and protects cells against apoptosis [18]. Bax is a pro-apoptotic inducer of Bcl-2 family proteins and forms heterodimers with a number of homologous anti-apoptotic proteins (Bcl-2, Bcl-X, and Bad), which are then inactivated to accelerate apoptosis [19]. Apoptosis is regulated through the balance between pro- and anti-apoptotic proteins. In the present study, expression of Bcl-2 and Bax was detected. We observed that Bcl-2 expression was markedly down-regulated in the As-ODN-hTR and ATRA treatment groups, especially in the combination treated group, compared with the control group. However, the expression of Bax was similar in all groups. This result indicates that Bcl-2 is involved in the apoptosis induced by As-ODN-hTR and ATRA, whereas Bax is not a direct target of these treatments. Recent studies have demonstrated that Bcl-2 is a key regulator for both the retinoic acid-induced apoptotic cell death and the telomerase activity. ATRA-induced cell differentiation is proposed to affect Bcl-2 mRNA stability indirectly by down-regulating the cellular levels of nucleolin, a potent Bcl-2 mRNA stabilization protein [20]. Also, it is increasingly recognized that *Bcl-2 *gene is involved in the regulation of telomerase activity induced by DNA-damage-related signals [21,22]. Consistent with these observations, close linkages between the administration of ATRA and the expression of Bcl-2, the telomerase activity and the expression of Bcl-2 were observed in the present study. Based on this observation, it was proposed that Bcl-2 is an essential factor and plays an important role in the apoptosis induced by ATRA and a decrease in telomerase activity.

## Conclusion

Collectively, the results of the current *in vivo *study demonstrate that in the combination of As-ODN-hTR and ATRA results in a synergistic anti-tumor effect. This anti-tumor effect may be mainly attributed to apoptosis induced by decreased telomerase activity. Bcl-2 is involved in this process, and appears to play an important role. Therefore, these findings suggest that the combination of As-ODN-hTR and ATRA may be a potential approach for the treatment of human oral squamous cell carcinoma.

## Competing interests

The authors declare there are no competing interests.

## Authors' contributions

WC was the PI of these experiments and was responsible for the study design, interpretation of the data and revision of the manuscript. QX was responsible for data acquisition, analysis of the work presented and the preparation of the manuscript. ZZ supervised the *in vivo *studies and was responsible for the study design. PZ participated in the study design and analysis of the growth inhibition data. All authors read and approved the final manuscript.

## Pre-publication history

The pre-publication history for this paper can be accessed here:


